# Improving Nitrogen Use Efficiency in Rice for Sustainable Agriculture: Strategies and Future Perspectives

**DOI:** 10.3390/life12101653

**Published:** 2022-10-20

**Authors:** Bo Wang, Genyou Zhou, Shiyang Guo, Xiaohui Li, Jiaqi Yuan, Anyong Hu

**Affiliations:** 1 Department of Food Crops, Jiangsu Yanjiang Institute of Agricultural Science, Nantong 226012, China; 2 School of Geographic Sciences, Nantong University, Nantong 226019, China; 3 Institute of Botany, Jiangsu Province and Chinese Academy of Sciences, Nanjing 210014, China

**Keywords:** rice, nitrogen, nitrogen use efficiency, agronomic practices, N efficiency-related genes

## Abstract

Nitrogen (N) is an important nutrient for the growth and development of rice. The application of N fertilizer has become one of the inevitable ways to increase rice yield due to insufficient soil N content. However, in order to achieve stable and high yield, farmers usually increase N fertilizer input without hesitation, resulting in a series of problems such as environmental pollution, energy waste and low production efficiency. For sustainable agriculture, improving the nitrogen use efficiency (NUE) to decrease N fertilizer input is imperative. In the present review, we firstly demonstrate the role of N in mediating root architecture, photosynthesis, metabolic balance, and yield components in rice. Furthermore, we further summarize the current agronomic practices for enhancing rice NUE, including balanced fertilization, the use of nitrification inhibitors and slow-release N fertilizers, the split application of N fertilizer, root zone fertilization, and so on. Finally, we discuss the recent advances of N efficiency-related genes with potential breeding value. These genes will contribute to improving the N uptake, maintain the N metabolism balance, and enhance the NUE, thereby breeding new varieties against low N tolerance to improve the rice yield and quality. Moreover, N-efficient varieties also need combine with precise N fertilizer management and advanced cultivation techniques to realize the maximum exploitation of their biological potential.

## 1. Introduction

**Figure 1:**
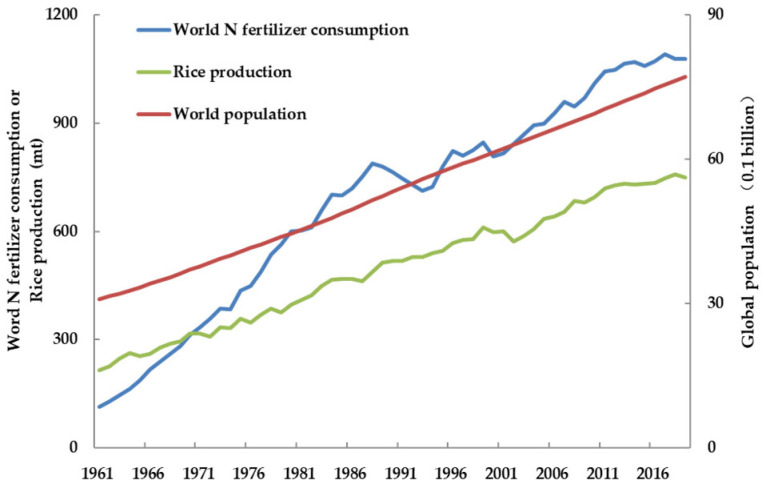
Temporal patterns of global rice production, human population growth, and nitrogen (N) fertilizer consumption, 1961–2019. Rice production and world population data were obtained from the Food and Agriculture Organization of the United Nations (FAOSTAT; http://www.fao.org/faostat/en/#data/OA accessed on 12 June 2022). Global N fertilizer consumption data were obtained from the International Fertilizer Association (IFA statistics, 13 July 2022; https://www.ifastat.org/databases/graph/1_1 accessed on 12 June 2022).

Rice, a grain crop, is the principal food source for more than half of the world’s population [1,2]. Due to expansion of cultivation areas, the introduction of new cultivars, and the use of chemical fertilizers, rice yield has been increased during the past 50 years, keeping pace with the world’s population growth (Figure 1) [3]. Nitrogen is one of the main nutrient elements required by rice. However, soil N content in farmland cannot maintain the requirements of rice growth and development. Therefore, the application of N fertilizer has become one of the inevitable ways to increase rice yield. During the past half century (1961–2010), the total grain production of China increased more than three-fold to achieve 480 million tons per year. At the same time, the application rate of chemical N fertilizer increased nearly 37-fold to reach 30 million tons of pure nitrogen per year, accounting for about one-third of the N applied globally [4]. In China, the average application rate of N fertilizer has reached 180 kg/hm^2^, which is 75% higher than the world average. However, the NUE is only 28%~35%, which is 15%~20% lower than that of the global average NUE [5,6]. Therefore, low NUE has gradually been becoming a prominent problem limiting rice production.

Nitrogen (N) is a crucial component for the synthesis of nucleotides, amino acids, and chlorophyll [7]. The introduction of semi-dwarf and high-yielding rice cultivars that respond to fertilizer inputs has highlighted the importance of N fertilization for rice cultivation since the Green Revolution. Nitrogen deficiency is one of the most common problems limiting rice growth, development, and production owing to poor N availability. The most basic physiological processes connected to biomass production and grain yield are affected by N availability, absorption, and utilization. The development of photosynthetic capacity and activity, the maintenance of photosynthetic activity, the establishment and maintenance of sink capacity (number and size of seeds), and agricultural product quality are the four key functions of N in biomass and the grain production of cereals [8]. As a result, N is an essential nutrient for agriculture and the security of global food supply, and N fertilizers are required to produce sufficient food to feed the increasing human population [9].

The efficient employment of N in agricultural production is crucial for yield and quality, environmental safety, and economic concerns [10]. The use of N fertilizers is a critical factor in maximizing yield potential in agricultural production, but has serious impacts on the environment, including high energy consumption, greenhouse gas emissions, and eutrophication of the water supply [9]. The NUE of rice is extremely low, particularly in irrigated ecologies, due to ammonia volatilization, denitrification, surface runoff, and leaching [11,12]. To increase plant NUE and decrease N fertilizer input in agricultural practice, it is crucial to understand the mechanisms of rice growth and development in diverse N-availability environments [13]. Therefore, increasing NUE is important to increase agricultural yields, to reduce production costs, and maintain environmental quality [14,15]. 

## 2. Nitrogen Functions and Nitrogen Deficiency Symptoms

Rice plants undergo a shift from vegetative to reproductive growth to produce a harvestable crop. Nitrogen is the primary component responsible for overall crop growth and development because it increases the number of leaves, individual leaf area, leaf area index (LAI), number of internodes, and internode length. N deficiency symptoms are first found in old leaves, and eventually become chlorotic and die [1]. N deficiency will further limit crop growth and development, resulting in premature senescence and decreased yields [2]. Nitrogen nutrition and its remobilization affect the number of grains per culm in cereals [16,17]. The nitrogen absorbed by roots is delivered from the roots to the upper leaves for plant development. The reallocation of N from older, senescing leaves to younger leaves and reproductive organs occurs in vegetative development advances [18]. 

### 2.1. Role of Nitrogen in Rice Photosynthesis

**Figure 2:**
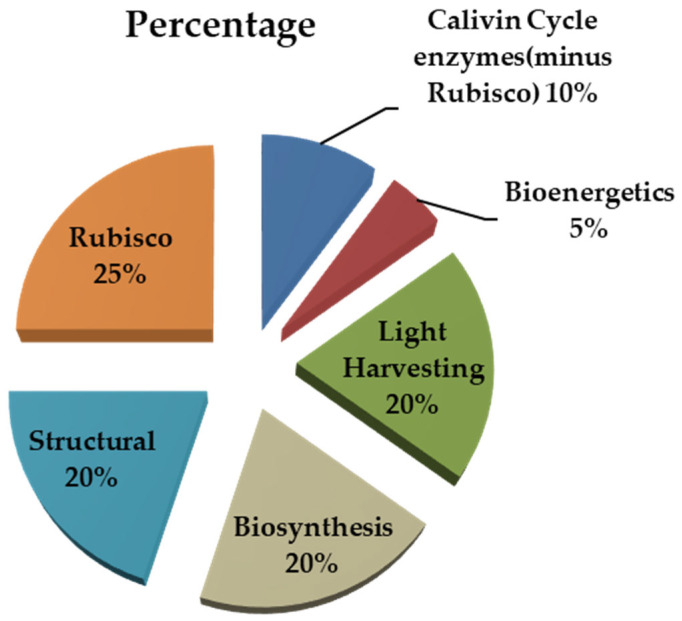
Partitioning of protein nitrogen in the leaf of a C_3_ plant.

Photosynthesis is vital for plant growth and crop yield [19,20]. Increasing the efficiency of photosynthesis is of importance for enhancing agricultural productivity and yield [21]. The photosynthetic NUE of rice is the highest among C_3_ species, indicating that rice assimilates a high amount of N per plant at the leaf level [1]. In photosynthesis, the ‘physical energy’ of photons is transformed into the ‘chemical energy’ of ATP and reduced metabolic intermediates (chiefly NADPH), which are utilized in the synthesis of carbon (C) and N assimilates, as well as carbohydrates and amino acids [22]. These fuels are used for the synthesis of biochemical components and ultimately supplied to plant organs. Because photosynthesis accounts for more than half of the N consumption in the leaf and most of the remainder is indirectly associated with its photosynthetic function (Figure 2), thus, N availability has a considerable impact on photosynthesis [23]. 

Ribulose-1,5-bisphosphate carboxylase/oxygenase (Rubisco), an essential but rate-limiting component of photosynthesis, is central to the photosynthetic process [24,25,26]. Rubisco has long been considered an important storage protein [26]. It can be mobilized from senescing leaves for the accumulation of protein content in young leaves and the biosynthesis of storage proteins in cereals grains when N supply is limited [18]. The involvement of Rubisco in the rice–N economy is complex, as evidenced by the fluctuating equilibrium between the maintenance of CO_2_-assimilating ability and Rubisco remobilization in old leaves. Nitrogen deficiency will shorten the leaf life span and decrease the accumulation of the assimilation product, and the rates of senescence and loss of photosynthetic capacity are more rapid than those under abundant N. Conversely, the contents of Rubisco and soluble protein (per unit of chlorophyll) increase with the increase in plant N content. Therefore, extending the effective life span of leaves via N application or breeding is a tried-and-true method for increasing rice yield. The ratio of Rubisco to chlorophyll in rice is relatively stable across a certain range of N application, but is increased with the further increase in leaf N content. Furthermore, increased N supply has a more profound effect on the increase in the ratio of Rubisco to chlorophyll than on the ratio of ATP synthase to chlorophyll [24,27]. 

### 2.2. Role of Nitrogen in Rice Root

Nitrogen is taken up by the roots, and assimilates are transported from the overground tissues to the roots to support regular growth and development [28]. The root structure and functions influence the nutrient and water uptake of plants [29,30]. Root architecture and crop yield are strongly affected by N concentrations in the soil. Mild N deficiency leads to the elongation of the lateral roots and primary root, while severe or prolonged N deficiency will inhibit the primary root growth and total root length [9]. Available N can diffuse along a high-to-low concentration gradient from the soil solution to the root surface. Nitrogen uptake from the soil solution and particles can be enhanced by increasing the root surface area [31]. Soil N deficiency will facilitate the allocation of more nutrients to boost root development, which has a negative effect on shoot biomass and production. The NUE cannot be improved by only increasing the root biomass or root surface area alone. 

### 2.3. Role of Nitrogen in Rice Metabolism

The signals from ammonium, nitrate, and nitrogen-containing metabolites including aspartate, glutamate, and glutamine, as well as the signals from C metabolism, constitute a complicated regulatory network involved in the C–N interaction [32]. Additionally, these metabolic signals will interact with hormones such as cytokinin, which respond to N supply and regulate metabolism and development. Nitrogen availability significantly affects the distribution of assimilated C, such as organic acids, starch, and sucrose, as well as the underlying mechanisms that are regulated by N, including both transcriptional and post-translational controls [33]. Metabolite profile analysis indicated that low N treatment caused decreased concentrations of total sugars and organic acids in the leaves, and increased concentrations of total sugars, organic acids and free amino acids in the roots [28].

### 2.4. Role of Nitrogen in Rice Yield and Yield Components

The number of panicles or heads, the number of spikelets per panicle or head, the weight of 1000 spikelets, and the sterility or number of filled spikelets per panicle or head are the yield components that determine grain yield in cereal crops [21]. The number of panicles or heads is determined in the vegetative developmental phase. In the reproductive growth stage, the number of spikelets per panicle or head is determined, while in the spikelet-filling or reproductive growth stages, the weight of spikelet and spikelet sterility are determined. Therefore, a crucial strategy to boost grain output is to provide sufficient N for rice plants or cereal crops under different growth periods. By considering the yield components, the yield of a crop may be stated in the form of the following equation [34]:

Grain yield (Mg ha^−1^) = number of panicles m^−2^ × spikelets per panicle × percentage filled spikelets × 1000 spikelets weight (g) × 10^−5^ (1)

The timing of N availability is crucial for rice growth and development because it influences all yield components, including number of tillers, number of productive tillers, grain number per ear, 1000-grain weight, and grain N content. The uptake of N after anthesis accounts for approximately 50% of the final grain N content in rice [35]. The rice productivity is mainly affected by the number of effective tillers rather than the total number of tillers. The number of productive tillers is influenced by environmental factors, particularly soil N concentrations during the early phases of tiller bud formation [36]. Adequate N supply is crucial for the grain development of rice and the increase in filled grains per panicle [37].

## 3. Definitions and Estimation of Nitrogen Use Efficiency in Plants

The measurement of NUE is important for assessing the fates of applied chemical fertilizers and their contribution to increasing crop yields [1,38]. Numerous definitions of NUE have been published in some studies (summarized in Table 1) [10,31,39,40,41]. In these definitions, plant productivity is considered in terms of ultimate yield, biomass, protein content, or a combination of these indicators. The formula of NUE calculation, i.e., NUE = NUpE × NUtE, includes N uptake efficiency (NUpE) and N utilization efficiency (NUtE). NUpE is defined as the plant N uptake divided by the N application rate. The NUtE is calculated as the ratio of the total grain yield to the total N contents of the plant.

## 4. Approaches to Improve Nitrogen Use Efficiency in Rice

Sustainable agricultural development requires taking advantage of advanced agronomic practices to ensure the more efficient use of N fertilizers, and thus reducing environmental pollution risks. Three main factors that influence the use efficiency of fertilizer N are crop demand, supply ability of plant-available N from the soil and fertilizer, and N loss from soil–plant systems [12,31]. In the last few decades, a large number of N management techniques have been used to increase crop production and NUE, including balanced fertilization, the use of nitrification inhibitors and slow-release nitrogenous fertilizers, the split application of N fertilizer, root zone fertilization, and so on. These N management practices will better accord with the N fertilizer requirement of the crop during the growth period, which can contribute to the improvement of NUE. Furthermore, due to high-efficiency selection and the pyramiding of genes, molecular breeding technology has been widely used to conduct the genetic improvement of rice varieties with high NUE genes [42,43].

### 4.1. Improvement of Nitrogen Use Efficiency: Physiological Perspective

Rice NUE is closely related to N uptake, N accumulation, and transport [38]. The morphological traits and activities of rice roots significantly affect the uptake and utilization of N fertilizer. The roots’ characteristics, such as larger root biomass, deeper root distribution, longer root length, greater root length density, and root oxidation activity contribute to higher NUE and crop yield [44]. Therefore, identifying the main root characteristics of high NUE in rice can provide references for the implementation of N application technology. Photosynthesis is the main source of assimilation products in rice. Stronger photosynthetic capacity, and the more efficient synthesis of photosynthetic products can lead to the accumulation of dry matter. The N content in leaves affects photosynthetic pigment content and the content and activity of Rubisco, which further affects the leaves’ photosynthetic capacity and efficiency. During the past few decades, rice breeding for high yield and rational water and fertilizer management have contributed to a substantial increase in rice yield per unit area [45]. Furthermore, the densities and methods of crop planting have reached optimization. Therefore, improving the utilization rate of light energy will be the main way to increase crop yields in the future because there is a good correlation between plant NUE and photosynthetic NUE [46]. Therefore, increasing the photosynthetic NUE of leaves will contribute to the increase in NUE and the yield of crops. Future research directions should focus on investigating the key factors of limiting photosynthesis and photosynthetic NUE, and explore effective ways to enhance photosynthesis and photosynthetic NUE.

### 4.2. Improvement of Nitrogen Use Efficiency: Agronomic Perspective

An appropriate N management strategy is essential to increase NUE and crop yields [8,47]. Scientific fertilization mainly includes the selection of suitable N fertilizer type (right source), an adequate application rate of N fertilizer (right rate), the appropriate timing of N application (right time), and the optimal position of N application (right place). The 4R nutrient management principles can be regarded as guidelines to improve traditional agronomic practices [8]. Types of N fertilizer have been updated rapidly for the past few years. Large granular fertilizers, slow-released fertilizers, compound fertilizers, formulated fertilizers, and fertilizers with various inhibitors have been gradually applied in rice production to effectively increase NUE through controlling the rates of dissolution and the release of fertilizers. The appropriate dosages of N fertilizer are mainly confirmed by the soil-testing formula fertilizer method, which can not only greatly enhance NUE and crop yield, but reduce the loss of reactive N [48]. The appropriate timing of N application can effectively increase NUE and crop yield, mainly by reducing the application rate of basal fertilizer and increasing the times of N applications in the later stages of crop growth [48]. Suitable fertilizer placement has indicated that the N fertilizer is applied in the locations where it can be easily absorbed and utilized by the crop root system. The deep application of N fertilizer is one of the simple and effective ways to improve NUE.

New N fertilizer species can significantly reduce N fertilizer loss, but higher production costs and sales prices seriously restrict their popularization and application. In the future, soil-testing formula for fertilization with N, phosphorus and potassium must be used to improve crop yield and fertilizer utilization. Cultivated land is dominated by small fields for rice planting. Therefore, it is essential to develop economically feasible mechanical tools and promote the popularization and application of deep fertilization, which will greatly improve NUE and crop yields.

### 4.3. Improvement of Nitrogen Use Efficiency: Genetic Perspective

Using N fertilizer excessively will not only make it difficult to continue to increase crop yield, but also reduce fertilizer use efficiency, resulting in the deterioration of the ecological environment. There are a large number of transporters involved in N uptake and utilization which play an important role in determining rice yield and quality. It is of great significance to investigate the mechanisms of N uptake, transport, and assimilation processes in rice, and to explore the genes of highly efficient N utilization for reducing both N fertilizer input and the cost of rice production, and increasing sustainable agricultural development.

Therefore, we provide a thorough summary of the currently reported genes on NUE, paying particular attention to N uptake, assimilation, and transport, as well as transcriptional control of N signaling in rice (Table 2). Thus far, N efficiency-related genes have been successively isolated or cloned at the molecular level, and their expression level, characteristic analysis, and functional identification have made great progress in the field of molecular biology. These genes related to N uptake, transport, or utilization efficiency include NRT/PTR (nitrate transport, peptide transport) genes, AMT (ammonium transport) genes, NPL family genes, and other types of genes.

#### 4.3.1. NRT Genes Involved in Rice NUE

Nitrate is not only the main inorganic N source in plants, but also acts as signal molecules to activate the expressions of a series of genes, thus improving NUE. Nitrate transporter 1 (NRT1)/peptide transporter (PTR) exist widely in plants. They are generally known as the NPF family due to high homologous sequences. Several studies have reported that the proteins encoded by NPF family genes have some functional commonalities, such as OsNPF2.2, OsNPF2.4, OsNPF4.5, OsNPF7.2, and OsNRT1, belonging to low-affinity nitrate transporters. The knockdown or overexpression of *NPF* genes affects nitrate uptake, distribution, and long-distance transport from root to stem in rice [52,53,54,55]. *OsNRT1.1B* is a NRT1 family gene localized at the plasma membrane which is induced by high nitrate concentration. There is a variation between threonine and methionine at position 327 of OsNRT1.1B which is specific to indica and japonica, thus resulting in the different absorption capacity for nitrate in them. The NIL family with indica *OsNRT1.1B* showed higher yield and N use efficiency. The variation in *OsNRT1.1B* greatly explains the difference in nitrate utilization between indica and japonica, and has potential utility value for improving NUE in japonica [56]. 

In addition to NRT1, NRT2 mainly participates in nitrate uptake and transport, and is an important nitrate transporter. OsNRT2.3A and OsNRT2.3B, as two members of the high-affinity nitrate transporter family, are two transcripts produced by *OsNRT2.3* during alternative splicing and are mainly expressed in the phloem of the plant. *OsNRT2.3a* encodes a phloem protein at the amino-acid position 516, responsible for long-distance nitrate transport from root to stem [57,58]. *OsNRT2.3B* encodes a phloem plasma membrane protein at amino-acid position 486, which maintains intracellular pH buffering capacity to increase nitrate uptake [59]. OsNRT2.4 is a plasma membrane-localized dual-affinity nitrate transporter required for lateral root formation and NO_3_^-^-N distribution in the shoots [60]. The nitrate transport ability of OsNRT2.1, OsNRT2.2 and OsNRT2.3a depends on their cooperation with OsNAR2.1 [61].

#### 4.3.2. The AMT Genes Involved in Rice NUE

The main N sources of plant uptake are ammonium and nitrate. Plants prefer to absorb NH_4_^+^-N than NO_3_^-^-N because the absorption and assimilation of NH_4_^+^-N require less energy. However, excessive NH_4_^+^-N uptake will cause ammonium toxicity to plants, so ammonium uptake and metabolism are strictly regulated by ammonium transporter (AMT). In recent years, a total of 10 *OsAMT* genes have been identified in rice by genetic engineering and forward genetics methods. According to their homology, they are divided into five classes: *OsAMT1* (*OsAMT1.1*, OsAMT1.2, OsAMT1.3), *OsAMT2* (*OsAMT2.1*, OsAMT2.2, OsAMT2.3), *OsAMT3* (*OsAMT3.1*, OsAMT3.2, OsAMT3.3), *OsAMT4* and *OsAMT5* (*OsAMT5.1*, OsAMT5.2).

Thus far, studies mainly focus on the three genes of the rice *OsAMT1* family, which shows a high homology of amino acid sequences and high affinity for NH_4_^+^-N uptake [62,63]. OsAMT1.2 and OsAMT1.3 are specifically expressed in the roots and are induced by ammonium [62]. *OsAMTl.1* is expressed in both root and shoot, and endogenous glutamine has a feedback regulation effect on it [64]. Furthermore, OsAMT1;1, OsAMT1;2 and OsAMT1;3 regulate the synergistic uptake of NH_4_^+^-N by rice under low-N conditions. The growth and N accumulation of single gene mutants exposed to low NH_4_^+^-N were not affected. The stem growth and N content of the *amt1;1/1;2* double-mutant decreased by 30%, while the growth of the *amt1;2/1;3* double-mutant was not affected. The *amt1;1/1;2/1;3* triple-mutant had the most significant phenotype, with a 59% inhibition of shoot growth and a 72% reduction in N accumulation [65].

#### 4.3.3. The NLP Family Genes Involved in Rice NUE

At present, several genes related to NUE have been identified in the NLP (nodule inception-like protein) family. Six *NLP* genes, named *OsNLP1*-*OsNLP6*, were identified in rice based on genomic data analysis [66]. Recent studies have shown that *OsNLP1, OsNLP3* and *OsNLP4* could directly bind to the promoter regions of many N-uptake and assimilation genes, then coordinate the expression of these genes, and thus promote N uptake and utilization. The expression of *OsNLP3* and *OsNLP4* were induced by low NO_3_^-^-N, but most of the proteins were localized in the cytoplasm and few were localized in the nucleus; thus, the expression of some N uptake and metabolism genes was weakly up-regulated [67,68]. The expression of *OsNLP1* was also induced by N starvation, but it was specifically localized in the nucleus and constitutively regulated N uptake and metabolism [69]. Under high NO_3_^-^-N conditions, nitrate facilitated the transport of *OsNLP3* and *OsNLP4* from the cytoplasm to the nucleus, and strongly upregulated the expression of N uptake and metabolism genes, so promotes N uptake and utilization [67,68]. However, when NH_4_^+^-N was used as the sole N source, a small amount of OsNLP4 proteins was localized to the nucleus in a much slower manner, and regulated N utilization together with *OsNLP1* [68].

OsNLP3 is a key transcription factor in nitrate signaling transduction and is regulated by phosphate signaling repressor SPX4. Under low NO_3_^-^-N conditions, SPX4 formed a complex with NLP3 which prevented NLP3 from entering the nucleus, and then inhibited the expression of N-responsive genes and N utilization. For high NO_3_^-^-N conditions, nitrate can promote the formation of an NRT1.1B–SPX4–NBIP1 complex. The ubiquitin ligase NBIP1 accelerated the ubiquitination and protein degradation of SPX4, and then released NLP3 into the nucleus, which activated the expression of N-responsive genes and the efficient utilization of N fertilizer [70].

#### 4.3.4. Other NUE-Related Rice Genes

In addition to those genes mentioned above, there are other genes involved in N uptake and transport in rice. The MADS-box family transcription factor plays an important role in the regulation of plant growth and development. *ANR1* is a key functional gene that has been verified to regulate lateral root development in *Arabidopsis* through the nitrate signaling pathway. Rice has five homologue genes of *ANR1*: *OsMADS23*, *OsMADS25*, *OsMADS27*, *OsMADS57*, and *OsMADS61*. Among these genes, OsMADS25, OsMADS27 and OsMADS57 are closely associated with N-signaling, which are mainly located at nucleus. They affected root architecture and NO_3_^-^-N accumulation by regulating the nitrate transporters [71,72,73].

During the growth and development of rice, three homologous genes encoding cytoplasmic glutamine synthetase are required, including *OsGS1;1*, *OsGS1;2* and *OsGS1;3*. *OsGS1;1* is mainly responsible for rice growth and grain filling [74]. *OsGS1;2* mainly affects plant height and the number of tillers and spikes [75]. In developing endosperm, NF-YC12 can directly bind with *FLO6* and the *OsGS1;3* promoter, thereby regulating starch and protein synthesis, respectively, and thus regulating the accumulation of seed storage materials [76].

Growth-regulating factor 4 (OsGRF4) is a key component of the GA signaling pathway and interacts with DELLA proteins. GRF4 is a positive regulator of the plant carbon–nitrogen metabolism, which can promote N uptake, assimilation, and transport, as well as photosynthesis, carbohydrate metabolism, and transport, but DELLA limits these processes. GA can promote the degradation of the DELLA protein, and then enhances the transcriptional activity of GRF4, so as to achieve the synergistic regulation of photosynthetic carbon fixation capacity in the leaves and N-uptake capacity in the roots, thus maintaining the balance of the C–N metabolism in plants [49]. *OsMYB61* regulates cellulose biosynthesis and NUE, and the introduction of indica *OsMYB61* into japonica varieties shows an effect of increasing yield, especially under N deficiency; its expression is controlled by GRF4 [77].

Recently, more and more N-efficient genes have been reported, such as *OsGOGAT1*, *OsTCP19*, *OsDREB1C,* and so on [78,79,80]. The significant value of these genes will contribute to improving N-uptake capacity, maintaining the N metabolism balance, and enhancing the NUE, thereby obtaining new varieties with low N tolerance to improve the rice yield and quality.

## 5. Conclusions

Nitrogen is an important element in plant growth, development, and yield formation. Improving plant NUE can efficiently reduce the application rate of N fertilizer and agricultural costs, thereby mitigating environmental pollution and ensuring food security. The agronomic strategies to improve NUE include screening rice varieties with high NUE, developing and applying new N fertilizers, optimizing the period and method of fertilization, scientifically using nitrification inhibitors and urease inhibitors, and popularizing and applying new technologies of fertilizer saving. These scientific and rational management measures of N fertilizer will contribute to realize food security and sustainable development in agriculture.

Improving the NUE of rice includes not only N uptake, but also N transport and the allocation between sink and source. At present, some progresses have been obtained for exploring the physiological and molecular mechanisms of highly efficient N utilization in rice. Recently, many NH_4_^+^- and NO_3_^-^-related transporters have been continuously identified in rice. Currently, the breeding of high NUE cultivars mainly relies on traditional breeding methods. Although many N-efficient genes have been identified, the successful application to crop production is still rare. Therefore, in the future, the breeding of N-efficient varieties with real practical value by using molecular marker-assisted breeding technology is still an important research topic.

In recent decades, with the development of multi-omics technologies, N uptake and utilization by rice have been systematically studied through transcriptomics, proteomics, and metabonomics, so as to improve the NUE of rice. In addition, it is necessary to further combine these results with precise field management and cultivation techniques to maximize the exploitation of the biological potential of new varieties.

## Figures and Tables

**Table 1:** Formulae and definitions of nitrogen use efficiency proposed for plants.

Number	Terminology	Formulae	Definition	Comments	Ref
1	Nitrogen use efficiency (yield)	NUE = NUpE × NUtE	Grain weight/N Supply (g per plant).	Consider yield only. High NUE crops will have a high yield but potentially a low N content in the biomass and in the grain.	[10,31,39]
2	Nitrogen uptake efficiency NUpE	UpE = Nt ÷ Ns	Nt = total nitrogen in plant, Ns = nitrogen supply (g per plant).	Measure efficiency of extracting N from soil.	[31,39]
3	Nitrogen utilization efficiency, NUtE	UtE = Gw ÷ Nt	Gw = grain weight, Nt = total nitrogen in plant.	Fraction of N converted to grain.	[31,39]
4	Agronomic efficiency	AE = (GwF − GwC) ÷ NF	NF = nitrogen fertilizer applied, GwF =grain weight with fertilizer, GwC =grain weight of unfertilized control.	Measures the efficiency of converting applied nitrogen to grain yield.	[31,39]
5	Apparent nitrogen recovery	AR = (NF uptake − NC uptake) ÷ NF × 100	NF uptake = plant nitrogen (fertilizer), NC uptake = plant nitrogen (no fertilizer), NF = Nitrogen fertilizer applie.	Measures the efficiency of capture of nitrogen from soil	[10,39,40]
6	Physiological efficiency	PE = (GwF − GwC) ÷ (NF uptake − NC uptake)	GwF = grain weight (fertilizer), GwC = grain weight (no fertilizer).	Measures the efficiency of capture of plant nitrogen in grain yield.	[39,40,41]
7	Fertilizer recovery efficiency	FRE = (GN − EN)/FN	GN = N removed in grain, EN = N from soil + rain, FN = fertilizer N applied.	Grain N from fertilizer as a fraction of that applied as fertilizer.	[39]
8	Fertilizer use efficiency	FUE = GY ÷ AF	GY = Grain Yield, AF = Applied Fertilizer.	Reflects the recovery of N in the crop as a fraction of fertilizer applied.	[39]

**Table 2:** Manipulation of genes to improve nitrogen use efficiency in rice.

Category	Functions	Gene	Technology	References
Nitrogen-uptake genes	Nitrate transporters	NRT2.1	Overexpression	[15,42,43]
		NAR2.1	RNA interference	[42]
		PTR9	Overexpression RNAi	[42,43]
		PTR6	Overexpression	[42,43]
		NRT1.1B	Overexpression	[42,43]
		NRT2.3a or NRT2.3b	Overexpression	[15,42,43]
		NRT1.1a or NRT1.1b	Overexpression	[42]
		NPF7.3 (PTR6)	Overexpression RNAi	[15,42,43]
		NPF7.7-1 or NPF7.7-2	Overexpression RNAi	[42,43]
		NPF6.1	Overexpression CRISPR/Cas9	[42]
		NPF4.5	Overexpression CRISPR/Cas9	[42,43]
	Ammonium transporters	AMT1.1	Overexpression	[15,42]
		AMT1-1	Overexpression	[42]
		AMT1;1	Overexpression	[42,43]
		AMT1-3	Overexpression	[15,42,43]
Nitrogen assimilation genes	Nitrate and nitrite reductases	NR2	Overexpression RNAi	[42]
	Glutamine synthetase	GS1;1 GS1;2	Overexpression	[15,42,43]
	Glutamate synthase	NADH- GOGAT	Overexpression	[15,42]
		AMT1;2 GOGAT1	T-DNA tagging+ crossing	[42]
	Glutamate dehydrogenase	GDH	Overexpression	[42]
Nitrogen remobilization and translocation	Asparagine synthetase	ASN1	Overexpression	[42,43]
	Alanine aminotransferase	AlaAT	Overexpression	[15,42,43]
		AAT1, AAT2, AAT3	Overexpression	[42]
	Amino acid transporters	AAP6a	Overexpression RNAi	[42]
		AAP3	Overexpression RNAi editing	[42,43]
		AAP5	Overexpression RNAi editing	[42,43]
		LHT1	Editing	[42]
		AAP4a or 4b AAP4	Overexpression RNAi editing	[42]
Transcription factors	--	CPK12	Overexpression	[42]
		ESL4	Overexpression	[42]
		GRF4	Overexpression RNAi editing	[42,43,49]
		NLP1	Overexpression editing	[2,42]
		myb61 grf4	Editing	[42]
		NLP4	Overexpression editing	[2,42]
		MADS25 MADS27 MADS57	Overexpressing	[39,50]
		MYB305	Overexpressing	[39,50]
		RDD1	Overexpressing	[15,50]
		BT	Repression expressing	[50,51]
		IDD10	Overexpressing	[50,51]
		NAP	Overexpressing	[39,50]

## Data Availability

The data presented in this study are available on request from the corresponding author upon reasonable request.
